# Study on Association between Spatial Distribution of Metal Mines and Disease Mortality: A Case Study in Suxian District, South China

**DOI:** 10.3390/ijerph10105163

**Published:** 2013-10-16

**Authors:** Daping Song, Dong Jiang, Yong Wang, Wei Chen, Yaohuan Huang, Dafang Zhuang

**Affiliations:** 1 College of Resource and Environmental Science, Nanjing Agricultural University, 6 Weigang Road, Xuanwu District, Nanjing 210095, China; E-Mails: songping_361@163.com (D.S.); chenwei@njau.edu.cn (W.C.); 2 State Key Laboratory of Resources and Environmental Information Systems, Institute of Geographical Sciences and Natural Resources Research, Chinese Academy of Sciences, 11A Datun Road, Chaoyang District, Beijing 100101, China; E-Mails: jiangd@lreis.ac.cn (D.J.); wangy@lreis.ac.cn (Y.W.); huangyh@lreis.ac.cn (Y.H.)

**Keywords:** metal mining area, heavy metal, land use, mortality

## Abstract

Metal mines release toxic substances into the environment and can therefore negatively impact the health of residents in nearby regions. This paper sought to investigate whether there was excess disease mortality in populations in the vicinity of the mining area in Suxian District, South China. The spatial distribution of metal mining and related activities from 1985 to 2012, which was derived from remote sensing imagery, was overlapped with disease mortality data. Three hotspot areas with high disease mortality were identified around the Shizhuyuan mine sites, *i.e.*, the Dengjiatang metal smelting sites, and the Xianxichong mine sites. Disease mortality decreased with the distance to the mining and smelting areas. Population exposure to pollution was estimated on the basis of distance from town of residence to pollution source. The risk of dying according to disease mortality rates was analyzed within 7–25 km buffers. The results suggested that there was a close relationship between the risk of disease mortality and proximity to the Suxian District mining industries. These associations were dependent on the type and scale of mining activities, the area influenced by mining and so on.

## 1. Introduction

Changes in world patterns of health, disease, and survival within populations over time indicate the interplay between human biology, culture, and environmental conditions [1]. While incidence and mortality rates for many diseases (cardiopulmonary and cerebrovascular diseases, cancer, respiratory system diseases, and digestive system diseases) are decreasing in the United States and many other Western countries, they are increasing in many less developed and economically transitioning countries because of adoption of unhealthy lifestyles, air pollution and worsening environmental factors such as heavy metal pollution [2], so most developing countries continue to be disproportionately affected by diseases related to infectious agents and contaminants in the soil, air, and water. With economic and mineral resources development, urban land use change continue to accelerate the replacement of land types, and also brings about many environmental problems, contributing to make heavy metal pollution currently a major environmental pollution problem. During the processes of mine exploitation and ore smelting, mine tailings, waste gases and wastewaters are created [3,4,5]. Heavy metals enrichment in the environment is associated with mining and human activity areas. Not only are mine workers directly affected by their work environment, but released toxic substances may also be transported from the mine sites and affect local communities and the environment [6,7,8,9]. Heavy metal ions in the environment can also be concentrated in organisms, which may further threaten human health through the food chain [10,11]. Some researchers have stressed that lifetime exposure to low levels of soil Cd could cause renal dysfunction [12,13]. Many sources of drinking and irrigation water have been contaminated by multiple heavy metals, such as cadmium and lead, with the advent of industrialization, particularly, in developing countries such as China [14].

With the progress of satellite and remote sensing technology like microwave remote sensing [15], optical remote sensing [16], hyper-spectral remote sensing [17], these and many other techniques have been used for mapping disturbances due to mining activities [18]. Integration of remote sensing with Geographical Information Systems (GIS) can further strengthen the capabilities of environmental impact assessment of mining activities at both the regional and local scales [19]. Some developed countries in Europe and America were successfully using hyperspectral techniques to investigate mine pollution as early as ten years ago. Spectral data from the AVIRIS instrument were used to evaluate mine waste and water pollution in USA [20,21]. In China, an object-oriented coal mining land cover classification method based on semantically meaningful image segmentation and image combination of GeoEye imagery and airborne laser scanning (ALS) data was presented [22]. Analyses were also conducted on the information features of a construction site, a cornfield and subsidence seeper land in a coal mining area with a synthetic aperture radar (SAR) image of medium resolution [23].

Many studies have suggested that heavy metals affect human beings’ health, and that there is a certain relationship between mortality and living near polluted areas [24,25,26]. However, the potential of remote sensing in conjunction with GIS largely remains largely underutilized in local mortality surveillance. Few studies incorporating geospatial tools to evaluate all the causes of death of a study area have been reported [27,28]. In our current study, we investigated the land use changes of Suxian District from 1985 to 2012, and explored the association between the disease mortality risk and distance to the Suxian District mining industries area. 

## 2. Materials and Methods

### 2.1. Study Area

**Figure 1:**
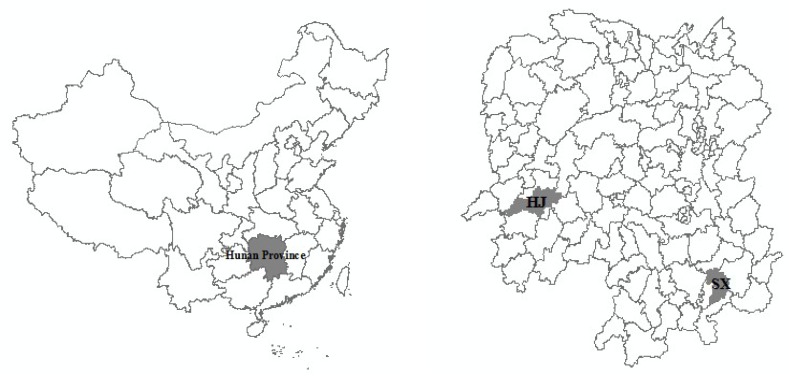
Geographical location of the study area. SX is Suxian District; HJ is Hongjiang County.

**Figure 2:**
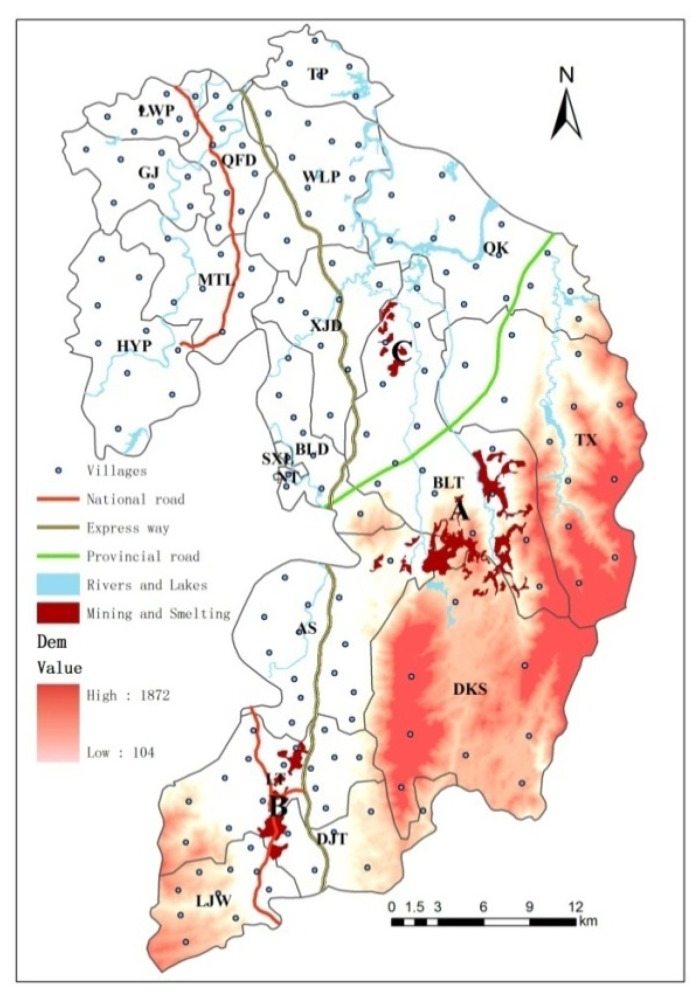
Suxian District, a metalliferous mining county in Chenzhou City, South China.

The study area, Suxian District, is located in Chenzhou City (Hunan Province, South China; Figure 1). Suxian District has a 1,000-year history of metal-mining activities and is characterized as a hilly and upland landscape. As of 2010, it comprises a total area of 1,346 km^2^, including 144.5 km^2^ of agricultural land and 744.2 km^2^ of forest land. The climate is subtropical with an average annual rainfall of about 1,500 mm. There are 172 administrative villages and 19 administrative townships (townships or streets) in the district, and 403,299 persons live there. There are three large nonferrous metal mines in the area: Shizhuyuan (W/Mo/Bi mine), Dongbo (Pb/Zn mine) and Manaoshan (Mn mine) around mine site A, the DJT smelter around the B ore smelting site, and BLT and DJT metals processing around the C machining site (Figure 2). The Shizhuyuan mine where Pb, Zn, W, Bi, Sn and Mo ores are mined and smelted is one of the largest metal mines in the world and is located 20 km from downtown. 

### 2.2. The Mining Industry Development

Suxian District is a world famous multi-metal mining area with a long history of mining activities, dating back to Jiaqing Period of the Qing Dynasty. In the late 1960s, Shizhuyuan in Suxian District was confirmed by exploration experts to contain highly diversified mineral varieties and large reserves, according to the results of identification of the Shizhuyuan metal ores. It was initially confirmed that there were 143 types of minerals in total, which represents a rare and exceptionally large ore deposit. Later other large metal mines in this region were also explored, such as the Dongbo Pb/Zn ore and Manaoshan Mn ore mines. However, affected by the backward mining technology, the development of the mining capacity and daily processing capacity were quite slow. The Shizhuyuan mine can be taken as an example. In 1980, its mining capacity was only 347,000 tons, while its mineral processing capacity was only 1,159 tons per day. Since the 1980s, the mining industry in Suxian District has developed rapidly, and the mining capacity and mineral processing capacity have multiplied. By 2010, its mining capacity had reached 3,500,000 tons, while its mineral processing capacity had reached 5,800 tons per day [29]. As a result, however, with the continuous expansion of the scale of mining, the frequent mining activities have become the major means by which heavy metals enter into the local environment and heavy metal pollution is increasingly more serious problem.

**Figure 3:**
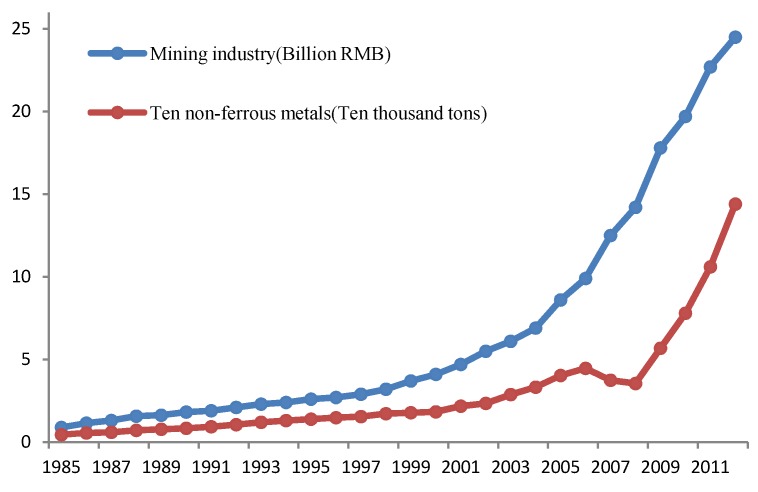
Profiles of the ten main non–ferrous metals by productions and values in Suxian district, 1985–2012.

As shown in Figure 3, the annual outputs of ten types of non-ferrous metals in Suxian District increased from the original 4,450 tons in 1985 to 144,000 tons in 2012 during the period of 1985–2012, a 31.4-fold increase. The value of the mining industry output increased from 0.089 billion RMB in 1985 to 2.45 billion RMB in 2012, a 26.5-fold increase. It is thus evident that the mining industry in Suxian District has developed rapidly in the recent 30 years [30].

### 2.3. Calculation and Statistic Analysis

#### 2.3.1. Remote Sensing Imagery

As the data source for extracting the land use types and depending on the availability of satellite data, data from the years 1985, 1995, 2000, 2005 were downloaded from the Landsat website (30 m resolution, USGS, Reston, VA USA; http://www.landsat.org), and we purchased the 2012 set of RapidEye images (5 m resolution, Kayser-Threde GmbH, Munich, Germany). All satellite data used were preprocessed using ENVI 4.7 (ITT VIS, White Plains, NY, USA). The same software was also used for generating the land use change maps. Visual interpretation of satellite data was done using ArcGis version 10.1 (ESRI, Redlands, CA, USA). The land uses could be divided into six types, which were cultivated land (CuL), forest land (FL), grassland (GL), water areas (WA), construction land (CoL) and unused (UL) land. The images of Suxian District were pre-processed using a multi-extraction method combining supervised and non-supervised classification. Using manual visual interpretation and computer technology node identification and extraction of land use information we eventually obtained land use maps for five periods. The transfer matrix method was applied to calculating land use changes with ArcGis10.1. 

#### 2.3.2. Retrospective Ecological Mortality

The Suxian District and the control area selected in this study, Hongjiang City, are both county-level administrative units in China’s Hunan Province. The two districts have similar geographical location, climate conditions and way of life. Their population sizes of both were more than 0.4 million. Hongjiang City, with its flourishing local planting industry, was honored as the “Wonderland without Pollution” by the World Health Organization. Suxian District, with its thriving mining industries, was honored as the “Hometown of China’s Non-ferrous Metals”.

The overall mortality data of the residents in the researched area and the control area during the period of 2008–2010 were obtained from the Centers for Disease Control (CDC) in China. The information included gender, age, type of work, address, descriptions of the cause of death and ICD10 codes [31]. The accuracy rate of the data was more than 95%. The mortality data were analyzed and compared to verify if there was a significant difference between the researched area and the control area. Taking the study of Neuberger and Fernández-Navarro [32,33] as reference, the overall mortality data were selected as the research priority. Because of the lack of the age composition of the rural population, the calculation results used for the mortality rate of residents in this study were all gross death rates. According to the ICD10 codes, the non-disease deaths were eliminated. The mortality rate formula was as follows:

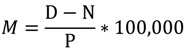

where *M* is the disease mortality rate; D is the total number of deaths; N is the number of deaths due to non-disease causes; P is the total number of population.

## 3. Result

### 3.1. Land Use and Mining Area Variation in the Researched Area during 30 Years

The variation of the area of six land-use types during the period of 1985–2012 in Suxian District is shown in Table 1. It was clearly seen that there were three different variation trends for the six land-use types. First, the areas of several types, such as forest land, grassland and unused land, decreased year by year. In 27 years, the forest land was reduced by 95.9 km^2^, which was 11.4% of the forestland area in 1985. The grassland decreased by 16.0 km^2^, or 11.9% of that in 1985. The unused land decreased by 14.6 km^2^, to 84.4% of the original area. Second, other areas were increasing year by year. For example, the land used for construction, accommodation, mining and industry increased by a total of 166.8 km^2^, which was 198.8% or almost twice the original amount of area. Third, some areas, such as the farmland and water areas, first increased and then decreased. However, the decreasing trend of the total farmland area played a dominant role, while there was little variation fluctuation of the increase and decrease of the water area.

**Table 1:** The land use situations in Suxian District (km^2^) by year.

Land use type	1985	1995	2000	2005	2012
CuL	215.3	228.1	214.4	201.3	177.6
FL	840.1	816.9	798.4	767.5	744.2
GL	134.1	133.9	125.5	122.8	118.1
WA	38.4	38.6	37.6	37.3	35.9
CoL	83.9	101.3	146.3	196.2	250.7
UL	17.3	10.3	6.9	4.0	2.6

Notes: CuL: Cultivated Land, FL: Forest Land, GA: Grassland, WA: Water Area, CoL: Construction Land, UL: Unused Land.

As shown in Table 2, the land area dedicated to large-scale mining and smelting factories in Suxian District presented an obvious rising trend. The land area increased from the original 7.09 km^2^ in 1985 to 21.13 km^2^ in 2012. The mining area, which was mainly distributed in the A, B and C subdistricts of Suxian District, thus expanded to three times the original size in 27 years. The number of large-scale mining and smelting factories first increased, reached a maximum of 22 in 2000, and then decreased. It has then stabilized at a certain amount and size in recent years.

**Table 2:** Mining area and number changes in Suxian district from 1985 to 2012 (km^2^).

	1985	1995	2000	2005	2012
Mining area	7.09	10.44	13.66	18.87	21.53
Number	14	17	22	20	18

### 3.2. Spatial Relationship between the Metal Mining Area and Mortality Rate of Residents

As shown in Table 3, the mortality rate of residents in the Suxian District in 2010 was 706 per 100,000, which was higher than the 613 per 100,000 in the control area of Hongjiang City. It was also higher than that of Chenzhou City and Hunan Province where Suxian District is located during the same period [34]. Meanwhile, the mortality rate of the residents of the Suxian District in 2010 was also higher than that of developed cities without mining industries, such as Beijing and Shanghai [35], so we think metal mines may be the main factor which caused the high mortality rate observed in Suxian District.

**Table 3:** Crude mortality in different regions in 2010 (per 100,000).

City	Suxian	Hongjiang	Chenzhou	Hunan	Beijing	Shanghai
Mortality	706	613	701	670	441	507

The disease mortality rates of the residents of villages and towns in the researched area and the control area were compared. It was found by the comparison that, as shown in Table 4, the range of the disease mortality rates of the residents of the researched area was higher than that of the control area. The lowest level of disease mortality rate was basically the same, but the maximum disease mortality rate of the residents in Suxian District was more than 1,000 per 100,000, which should draw our attention. Meanwhile, the mean value, median and standard deviation of the disease mortality rate of the residents of Suxian District were all higher than those in the control area by comparison. This indicated that the disease mortality rates of residents of each village and town in the Suxian District had an obvious variation difference. There was a large possibility that the deaths of residents were influenced by external factors. However, the variation difference was not obvious in the control area.

**Table 4:** Township population mortality of Suxian and Hongjiang in 2010 (per 100,000).

	Range	Mean	Median	Std. Dev
Suxian	342–1,048	654	695	1.656
Hongjiang	350–881	588	616	1.056

**Figure 4:**
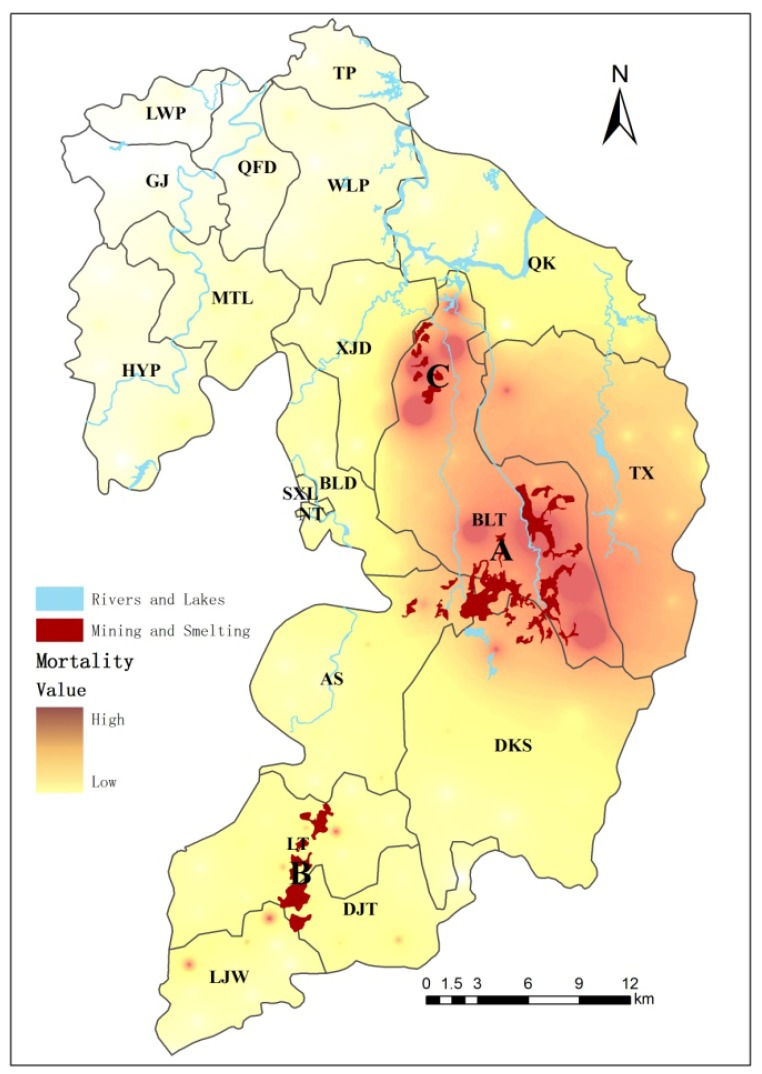
Geographical distribution of mortality levels in Suxian District of Chenzhou City, South China.

As shown in Figure 4, the disease mortality rate of the residents of Suxian District was analyzed spatially by the weighted inverse distance method with the Geographic Information System software ArcGIS10.1. Three hot spots with high mortality rates were found, which were distributed in mining area A (Shizhuyuan, Manaoshan and Dongbo), smelting factory area B (Dengjiatang) and around mining and smelting area C (Xianxichong), respectively. The mortality rates presented a decreasing trend in space with the increase of the distance from the mining and smelting area pollution sources. There were 73 villages in the whole Suxian District where the disease mortality rate of the residents exceeded the average value of 654 per 100,000. These villages accounted for 42.9% of the total number of villages. The villages with high mortality rates were highly concentrated within a 2 km range from the buffer area, and as the buffer distance increased, the percentage of the number of villages with high mortality rates decreased continuously. 

**Table 5:** Villages and population distribution in different buffers in Suxian District in 2010.

Buffer	Total villages		High mortality villages	
	Number	Population	Number *	Rate (%)
1 km	14	17,658	13	92.9
2 km	19	23,805	18	94.7
3 km	40	61,846	25	62.5
5 km	71	103,720	40	56.3
7 km	94	139,239	47	50.0
10 km	116	175,500	57	49.1
15 km	140	221,871	69	49.3
25 km	170	272,264	73	42.9

Buffer stands for the radius of different buffer of the mine area; Number stand for number of villages located in the buffer; Population stands for total amount of people in the buffer; Number * is number of villages with high mortality rate in the buffer; Rate equals to the ratio of Number * to Number.

When the distance from the buffer area was 7 km, the number of villages with high mortality rates accounted for 50% of that of the villages in the buffer area. At that time, the size of population in the buffer area had already exceeded half of the rural population in the Suxian District. When the distance from the buffer area exceeded 7 km, the proportion of the villages with high mortality rate had no obvious variation as the distance increased (Table 5).

**Figure 5:**
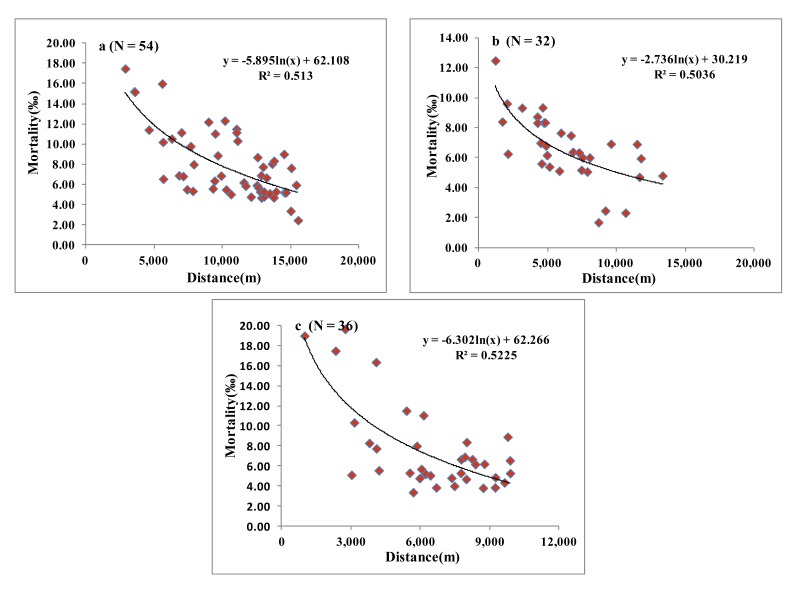
Relationship between mortality in Suxian District and distance from Shizhuyuan (**a**) DJT smelters (**b**) and Xianxichong (**c**) mining sites of Chenzhou City, South China.

As shown in Figure 5, the relationship between the mortality rate of the residents of villages and the distance from the mining area conformed to a linear correlation model. The mortality rate decreased with the increase of distance from the mining area. The variation trend of the mortality rate was not obvious in villages at the distance greater than the critical value of the model. Three models in Figure 5(a–c) were corresponded to three polluted areas, namely, A, B and C, respectively. The number (N) of villages matching the model correlation were 54, 32 and 36, respectively. The critical distances of the mining areas A, B and C were calculated by the model to be 15.5 km, 13.3 km and 9.8 km, respectively.

## 4. Discussion

### 4.1. Development of Mining Area and Variation of Land Use

The terrain in Suxian District is mainly hilly, with vast forest and grassland areas. There are numerous mountain ranges in the researched area, which were also the basic conditions of the existence of the metal mineral deposits. With the economic development and technical progress, the demand for non-ferrous metals is also growing. Especially after 2000, the scale of mining has developed rapidly, and the mining area developed rapidly due to the gradually expanding scale of mining and the resulting economic benefits. The facilities surrounded the mining area improved continuously. The Shizhuyuan mining area was taken as an example. Due to the development of the mining area, the demand for labor force increased and as a result, residential areas and supporting living facilities were constructed surrounding the mining area. Due to the improved living conditions, the number of residents has increased continuously in the area surrounding the mining activities, and the forest land and farmland were thus occupied even further. In short, the relationship between people and the mining industry was getting increasingly closer due to the continuous development of the mining areas.

### 4.2. Disease Mortality Rate of Residents and Spatial Distribution of Mining Area

First, it was known that the mortality rate of the residents of Suxian District was higher than that of the control area and other developed cities (Table 4 and Table 5). It was found that the geographic location and climatic conditions of Suxian District and control area were very similar. The pollution by heavy metals caused by mining areas on the surrounding environment had been reported in the 1990s [36,37]. In recent years, the Shizhuyuan polymetallic mine area in the Suxian District and the soil pollution and heavy metal pollution for plants in the surrounding farmland of the Dengjiatang smelting factories were investigated further by Zhai, Liu and Liao *et al.*, respectively. The results indicated that the content of heavy metals such as Cd, As, Zn, Pb and Cu in the soil, rice and vegetables in the areas exceeded the national standard [38,39,40]. Meanwhile, in the Hongjiang City control area where local crop farming was thriving heavy metal pollution rarely happened. The surrounding environments of mining activity areas in different countries and regions of the World were studied, and it was indicated that the effects of heavy metals on the environment, especially on water bodies, soil and plants are ubiquitous [41,42,43,44]. It was confirmed through numerous studies that mining activities exposed residents in mining areas to the heavy metals in the environment for a long time, which led to significant increases in cancer mortality rates [45,46,47,48,49].

The disease mortality rates of the residents in Suxian District were generally high in three mining areas. Within a certain distance, there was a decreasing trend for the mortality rate as the distance increased. This was similar to the spatial distribution features of Cd and As concentrations in the soil and plants in the Suxian District studied by Zhai and Liao *et al.* [38,40]. The buffer areas were established in the whole mining area of Suxian District. It was found that the percentage of the number of villages with high mortality rate decreased continuously with the increase of buffer distance. The villages with high mortality rate were highly concentrated within a 2 km buffer area range, which was also the area with high concentrations of heavy metals. When the buffer area distance was 7 km, the number of villages with high mortality rates accounted for 50% of that of the villages in the buffer area. The concentration of heavy metals also decreased correspondingly. At the same time, the size of the population in the buffer area had already exceeded half of the rural population in Suxian District. Because of the increase of population, the probability of the heavy metals impact also increased continuously. When the buffer area exceeded 7 km, the proportion of villages with high mortality rate had no significant variation with the increase of distance. This was consistent with the studies of Fernández-Navarro and Garcia-Perez *et al.* [33,50,51].

The relationship model of distance and mortality rate was analyzed separately for each mining area. It was found that the effect of each mining area on the mortality rate was in accord with a certain exponential model. When R^2^ = 0.5, the distance was taken as the critical distance. The critical distances of the three mining areas presented the following relationship, A > C > B. Although the minimum critical distance was 9.8 km, it was found that when the mortality rate was 654 per 100,000, the corresponding distances of three models were 12 km, 6 km and 7 km, respectively. Only the distance of mining area A was greater than 7 km. The other distances were equivalent to the critical distance in the buffer area. The possible reason was analyzed. It was concluded that there was a long mining history for mining area A, which had the largest mining scale and diameter. The varieties of heavy metal were complex. Because of the high altitude, the pollutants in mining area A flowed downstream in two major rivers, the East River and West River. The heavy metals were more easily constantly spread to the downstream area with the water, which increased the influence radius. Area B was taken as the historic ore smelting processing field , which was mainly distributed in the town of DJT. As and other heavy metalz were mostly associated with the metal mines of Pb, Zn and Cu. The pollutants generated in the industrial activities such as mining and smelting were emitted into the surrounding environment, therefore, the As concentration was extremely high [52,53]. As a result, the yield and quality of crops in the contaminated area was decreased. More than 300 people were hospitalized in 1999 due to the arsenic pollution, and 50 hm^2^ of paddy fields had to be abandoned because of the arsenic pollution. Although the government had consecutively closed the smelting factories in the area after 2000, the effects of heavy metals on the residents surrounding DJT still existed. This was because the incubation period of the toxic effect of heavy metals such as arsenic on human body can last several decades. The area and size of mining area C were the smallest. The heavy metal variety was not as abundant as that in mining area A. Figure 5(c) showed that the influence of mining area C on the mortality rate was small. However, the mining area C was located in the downstream regions of the East River and West River, so the possibility of pollution from water sources was greatly increased. As a result, the mortality rate in the mining area C was also significantly higher than that of other barren areas.

## 5. Conclusions

The land use trends obviously changed in the researched area over nearly 30 years, namely, during the 1985–2012 period. The forest land and farmland decreased on a large scale, while the construction land, the industrial and mining land increased greatly. The mining industry developed rapidly in the local area with the mining area increasing to three times the original area. The mining area was close to the urban area and the residential area, which were easily affected by the emissions of heavy metals by the mining and smelting activities.

An overall pattern of elevated disease mortality was observed in Suxian District. Three hotspot areas with high disease mortality were identified around the Shizhuyuan mine sites, Dengjiatang metal smelting sites, and Xianxichong mine sites. Suxian District disease mortality decreased with increasing distance from the mining and smelting areas. Population exposure to pollution was estimated on the basis of distance from town of residence to pollution source. We analyzed the risk of dying from disease mortality in a 7 km zone around the mining installations, and conducted individual analyses within a 25 km radius of each mine site. The results suggested an association between risk of disease mortality and proximity to Suxian District mining industries. These associations were dependent on the type, scale, area of the metal mine. We also should be concerned about the fact that the river and the elevation have an effect on the disease mortality.

A preliminary exploration was made on the relationship between the mining industry and the mortality rate in Suxian District in this study, which still has numerous deficiencies. The specific influencing factors (water sources, soils, crops and dietary habits) which affect the high and low incidence of specific disease mortality rates (such as the mortality rates due to different kinds of tumor) should be purposefully analyzed quantitatively through comparison in subsequent research. Thus, the main limiting factors influencing the mortality rate of the residents could be confirmed more accurately, which would provide a certain theoretical basis concerning city planning and health risks of residents in the future.
